# Characterization of a High Hierarchical Regulator, *PtrGATA12*, Functioning in Differentially Regulating Secondary Wall Component Biosynthesis in *Populus trichocarpa*

**DOI:** 10.3389/fpls.2021.657787

**Published:** 2021-04-21

**Authors:** Mengxuan Ren, Yang Zhang, Cong Liu, Yingying Liu, Shuanghui Tian, He Cheng, Huaxin Zhang, Hairong Wei, Zhigang Wei

**Affiliations:** ^1^Research Center of Saline and Alkali Land of State Forestry and Grassland Administration, Chinese Academy of Forestry, Beijing, China; ^2^State Key Laboratory of Tree Genetics and Breeding, Northeast Forestry University, Harbin, China; ^3^College of Forest Resources and Environmental Science, Michigan Technological University, Houghton, MI, United States

**Keywords:** *PtrGATA12*, hierarchical regulator, biological characterization, secondary cell wall, *Populus trichocarpa*, coordinated regulation

## Abstract

In plants, GATA transcription factors (*TFs*) have been reported to play vital roles in to a wide range of biological processes. To date, there is still no report about the involvement and functions of woody plant GATA *TFs* in wood formation. In this study, we described the functional characterization of a *Populus trichocarpa* GATA TF, *PtrGATA12*, which encodes a nuclear-localized transcriptional activator predominantly expressing in developing xylem tissues. Overexpression of *PtrGATA12* not only inhibited growths of most phenotypic traits and biomass accumulation, but also altered the expressions of some master *TFs* and pathway genes involved in secondary cell wall (SCW) and programmed cell death, leading to alternated SCW components and breaking forces of stems of transgenic lines. The significant changes occurred in the contents of hemicellulose and lignin and SCW thicknesses of fiber and vessel that increased by 13.5 and 10.8%, and 20.83 and 11.83%, respectively. Furthermore, PtrGATA12 bound directly to the promoters of a battery of *TFs* and pathway genes and activated them; the binding sites include two *cis*-acting elements that were specifically enriched in their promoter regions. Taken together, our results suggest *PtrGATA12*, as a higher hierarchical *TF* on the top of *PtrWND6A*, *PtrWND6B*, *PtrMYB152*, and *PtrMYB21*, exert a coordinated regulation of SCW components biosynthesis pathways through directly and indirectly controlling master TFs, middle-level *TFs*, and further downstream pathway genes of the currently known hierarchical transcription network that governs SCW formation.

## Introduction

Wood accounts for the bulk of biomass produced by land plants, and has been considered to be an important renewable and environmentally friendly source of bioenergy (Ohlrogge et al., 2009). The major components of wood, secondary cell walls (SCWs), consist of a cross-linked matrix of cellulose, hemicellulose, and lignin biopolymers (Zhong et al., 2010a). To genetically improve the productivity and quality of woods for better utilization, it is imperative to identify the molecular regulatory mechanisms associated with differential regulation of SCW biosynthesis. Studies in herbaceous and woody plants have revealed that SCW formation is regulated by a hierarchical transcription regulation network (hGRN) that is comprised of more than four layers of genes (Mellerowicz and Sundberg, 2008; Zhong et al., 2008, 2010a, 2011; Zhong and Ye, 2010; Taylor-Teeples et al., 2015; Rao and Dixon, 2018). The currently known top-layered regulatory genes in this network are NAC *SND1* (McCarthy et al., 2009), *VND6/7* (Ohashi-Ito et al., 2010; Yamaguchi et al., 2010), and *PtrWND2B*/*6B* (Zhong et al., 2010a, 2011), which function as master regulators for xylem cell differentiation (Zhong et al., 2010a, 2011). Downstream of these NAC master *TFs* are the *MYB46/83* and *PtrMYB3/20*, which function as intermediate level hub regulators (McCarthy et al., 2009, 2010; Ko et al., 2012), whose targets are SCW-associated genes, including other lower-level MYB *TFs*, such as *MYB58*, *MYB63*, *PtrMYB125*, *PpDof5*, and *PdOLP1* (Zhou et al., 2009; Rueda-Lopez et al., 2017; Balmant et al., 2020; Li et al., 2020). At the terminal are a variety of bottom-layered pathway genes responsible of SCW component biosynthesis (Zhong et al., 2010a). Although many regulatory genes have been identified, it is critically important to identify more complements of this hGRN. In addition, we are especially interested in identifying the hub regulators and the high hierarchical regulators. Hub regulators can take multiple “commands” from the higher hierarchical levels, synthesize new components, and then pass them down to biosynthetic pathways, whereas the high hierarchical regulators are those that are capable of regulating multiple pathways. For this reason, we initiated a project to identify more middle-level “hub” regulators, and high hierarchical regulators that can differentially control multiple SCW component biosynthetic pathways, and believe that they are instrumental for genetical engineering wood composition and quality.

GATA TF family, which is characterized by a type-IV zinc-finger motif with a CX_2_CX_17–20_CX_2_C domain followed by a basic region facilitating DNA binding the WGATAR (W = T or A; R = G or A) in the promoter region, is widely distributed in fungi, metazoans, and plants (Lowry and Atchley, 2000). A GATA zinc-finger TF with 17–18 residues in the binding loop is a characteristic feature of animal and fungal GATA TFs, while a plant GATA zinc-finger TF possess 17–20 residues (Reyes et al., 2004; Behringer et al., 2014). Plant GATA *TFs* have been identified in multiple plant species, such as *Arabidopsis thaliana* (Zhang et al., 2018a), *Oryza sativa* (Reyes et al., 2004), *Glycine max* (Zhang et al., 2015), *Gossypium raimondii* (Zhang et al., 2019), *Malus domestica* (Chen et al., 2017), and *Populus trichocarpa* (An et al., 2020). *GATA TFs* have been reported to play important roles in a wide range of biological processes of plants, such as vegetative growth and development (An et al., 2020), seed dormancy and germination, flowering and shoot apical meristem development (Behringer and Schwechheimer, 2015), chloroplast development (An et al., 2014; Zhang et al., 2015), cell elongation (Shikata et al., 2004), response to light and stress (Zhang et al., 2015; Kobayashi and Masuda, 2016), hormone signaling (Zhang et al., 2015; Kobayashi et al., 2017), and N metabolism (An et al., 2014). Besides these, it has been reported that overexpression of *AtGATA12* induces ectopic differentiation of xylem vessel elements and SCW deposition through activating *VND7* expression in *A. thaliana* (Endo et al., 2015). Since AtVND7 is a relatively high hierarchical master regulator of lignin and cellulose pathway regulator (Zhong et al., 2008), AtGATA12 is more likely to be a hierarchical regulator. Although the major transcriptional programs regulating wood formation are conserved in vascular plants, some variations of ortholog gene functions might have evolved since wood anatomy and compositions vary greatly among different plant species (Zhong et al., 2010a). For instance, *A. thaliana MYB103* preferentially induces the expression of genes for cellulose biosynthesis but not xylan and lignin (Zhong et al., 2008), whereas its poplar ortholog, *PtrMYB128*, is able to activate the genes related to entire SCW component biosynthesis (Zhong et al., 2011). In addition, *P. trichocarpa* PtrSND1-B1 mediates a four-layered hGRN while its counterpart in *A. thaliana*, AtSND1, mediates a three-layered hGRN (Chen et al., 2019), indicating that PtrGATA12 may mediates different target genes and a network especially when taking the more complicated wood components and the augmented wood formation in poplar into account, These facts taken together made us to choose GATA12 gene to study.

In this report, we demonstrated that *PtrGATA12* (Potri.006G237700), a *P. trichocarpa* ortholog of the *Arabidopsis GATA12*, had vital roles in poplar woody formation. We showed that *PtrGATA12* is prominently expressed in developing secondary xylem tissues, and is a transcriptional activator that functions in the nuclei. Overexpression of *PtrGATA12* in poplar leads to arrests of growths and development of most phenotypic traits, alternations of SCW component and thickness, and increase of breaking forces of stem, accompanied by the changes of expression of *TFs* and pathway genes related to SCW biosynthesis. Furthermore, we revealed that PtrGATA12 was capable of directly activating a number of poplar wood-associated *TFs* and pathway genes through binding to the *cis*-elements in their promoter regions. Our findings suggested that the *PtrGATA12*, as a higher hierarchical transcription activator, plays significant roles in activating hemicellulose and lignin biosynthesis of SCW through directly or indirectly regulating a number of *TFs* and pathway genes that are elements of the hierarchical transcription regulatory network of wood formation, and can be used to genetically modify wood characteristics.

## Materials and Methods

### Plant Materials

The plantlets of *P. trichocarpa* clone Nisqually-1, whose genome was sequenced (Tuskan et al., 2006) early, were obtained from the Shanghai Institute for Biological Sciences, Chinese Academy of Sciences, and vegetatively propagated in our lab using tissue culture (Li et al., 2017).

One-year-old *P. trichocarpa* trees were propagated and planted in a mixture of turfy peat and sand (2:1 v/v) and grown under 16 h/8 h day/night photoperiod at 25°C in the greenhouse at Northeast Forestry University for 90 days. The primary shoot leaves, transition leaves, secondary leaves, primary xylem, transition xylem, secondary xylem, primary phloem, transition phloem, secondary phloem, and roots, which were used to analyze the tissue-specific expression patterns of PtrGATA12, were collected and immediately frozen in liquid Nitrogen and stored at −80°C. The RNA was isolated according to a previously published method (Liao et al., 2004) and later treated with DNase I (Qiagen) to remove genomic DNA (Kolosova et al., 2004).

### Cloning *PtrGATA12* From *P. trichocarpa*

Five microgram total RNA from secondary xylem of *P. trichocarpa* stems was used for synthesizing cDNAs using SuperScript II Reverse Transcriptase (Invitrogen). The full *PtrGATA12* (Potri.006G237700) cDNA was amplified with gene-specific primers (Supplementary Table S1). The PCR products were cloned into *pMD18-T* vector (TaKaRa), and then transformed into *Escherichia coli* cells (DH5α) for validation by Sanger sequencing.

### Subcellular Localization

The full-length coding region of *PtrGATA12* without termination codon was amplified using specific primers (Supplementary Table S1) and then fused to the N-terminal of green fluorescent protein (GFP) driven by *CaMV 35S* promoter in *pGWB5* vector. The two fusion constructs were delivered into onion epidermal cells *via* particle bombardment (GJ-1000). The GFP fluorescent images were photographed with confocal microscopy (Leica TCS SP5) at 24 h after bombardment.

### Transcriptional Activation Assay

The transcriptional activation of PtrGATA12 on putative targets was corroborated using the yeast two-hybrid system. The complete CDS of *PtrGATA12* was amplified using specifically designed primers (Supplementary Table S1). The amplified fragments were fused in-frame to the pGBKT7 vector to generate the pGBKT7-*PtrGATA12* construct. The pGBKT7-*PtrGATA12* and the *pGBKT7* blank vector (as negative control) were transformed into AH109 yeast cells independently. The transformed AH109 yeast cells were plated onto SD/-Trp (growth control), SD/-Trp/-His/-Ade, and X-α-Gal media and incubated on at 30°C for 3–5 days to identify the transcriptional activation.

### Transformation of *P. trichocarpa* for Generating PtrGATA12 Transgenic Lines

The *PtrGATA12* was amplified with specific primers (Supplementary Table S1), and then inserted into the pROKII vector. The pROKII-*PtrGATA12* was first transferred into *Agrobacterium tumefaciens* EHA105 using the freeze-thaw method. The transgenic method was described below based on previous study (Li et al., 2017). The genomic DNA of all kanamycin-resistant shoots amplified by regular PCR using the PROKII sequencing primers listed in Supplementary Table S2 to verify whether *PtrGATA12* was integrated into poplar genome.

All tested *PtrGATA12* transgene lines, and wild-type (WT) poplar were propagated and planted in a mixture of turfy peat and sand (2:1 v/v) and grown under 16 h/8 h day/night photoperiod at 25°C in the greenhouse. When the *PtrGATA12* transgene lines grown for 90 days, they were subsequently used for further characterization.

### Phenotypic Trait Measurement

The developmental stages of tissues were standardized by employing a plastichron index (PI; PI = 0 was defined as the first leaf greater than 5 cm in length; PI = 1 was the leaf immediately below PI = 0). Stems spanning PI = 5 to PI = 8 were cut and frozen in liquid nitrogen, and retained for *PtrGATA12* transcript abundance and transcript abundance of the known TFs and pathway genes related to fiber formation, SCW biosynthesis, and PCD analysis. Stems spanning PI = 8 to PI = 10 were cut and used for SCW thickness, breaking force, and cell wall chemical composition analyses. The internode lengths were determined by distance between two adjacent leaf petioles along the stems. Leaves from PI = 4 to PI = 6 were measured for lengths and widths.

Before harvesting, we measured the height of each plant from the root to the tip of the tallest bud and the base diameters above ~3 cm of soil to calculate its biomass. The fresh weight is determined immediately after harvesting the whole plant. Then, the plant material was placed into the oven and incubated at 100°C for 10 min, and then at 75°C until the weight did not change. The final unchanged weight was recorded as the dry weight.

### Determination of Break Forces

The breaking force, which has been reported to be correlated with the cellulose content in stem of maize (Dhugga, 2007), refers to the tensile or bending strength used to break stem. The breaking forces of stem segments were analyzed using YYD-1 plant stalk analyzer according to the manufacturer’s instructions (Zhejiang Top Instrument Co., Ltd.).

### Scanning Electron Microscopy

Stem segments were prepared by freeze-drying for scanning electron microscopy (SEM; S-4800, HITACHI). Dry segments were mounted on aluminum stubs using carbon tape with conductive silver paint applied to the sides to reduce sample charging. The segments were then sputter-coated with gold in an E-100 ion sputter. Imaging was performed at beam accelerating voltages from 12.5 to 15 kV. The secondary wall thicknesses of fibers in the SEM micrographs were quantified in a randomly selected area of 45 cells using Image J software.^1^

### Histological Analysis

The 90-day-old poplar basal stems were cut into 0.5 cm fragments and immersed into 4% paraformaldehyde at 4°C for 3 days, washed twice in 1x PBS for 15 min, and then dehydrated in a graded ethanol series (2 h each time). The stem fragment was then incubated in xylene: ethanol 1:3, 2:2, 3:1 in sequence, and then incubated in 100% xylene twice for 2 h each time. Following that, the stem fragments were incubated overnight in dimethylbenzene: paraplast 3:1 at 63°C, and then transferred into pure paraplast for an overnight treatment at 63°C.

The stem fragments (0.5 cm long) embedded in the paraplast were cut into stem sections (1 μm thick) with a Leica EM UC6 microtome, and then stained with 0.01% Calcofluor White, and the cellulose was observed with an inverted UV fluorescence microscope. Under this condition, only the secondary walls show bright fluorescence. At the same time, the stem sections, which were cut into 50 μm thick, were stained with phloroglucinol-HCl for observing the lignin, which takes on bright red under an optical microscope. To examine the xylan contents, stem sections (1 μm thick) were probed with LM10 monoclonal antibodies, which are capable of binding to 4-O-methylglucuronoxylan, and detected with fluorescein isothiocyanate-conjugated secondary antibodies. The fluorescence-labeled xylan signals were visualized and imaged with an Olympus DX51 light microscope.

### Determination of Content of Cellulose, Hemicellulose, and Lignin

The determination of the contents of lignin, cellulose, and hemicellulose was conducted by following a previously published method (Liu et al., 2017) with a ANKOM 2000i Automatic fiber analyzer (Ankom).

### Gene Expression Analysis of Poplar

Five microgram total RNA from xylem of stems spanning PI = 5 to PI = 8 of *PtrGATA12* transgenic lines was used for synthesizing cDNA. Samples of cDNA were run in triplicate in an Applied Biosystems 7,500 Real-Time PCR System to determine the critical threshold (Ct) with the SYBR premix ExTaq kit (TaKaRa).

Primers used for quantitative RT-PCR (qRT-PCR) of the Potri.001G188500, Potri.006G237700, Potri.018G044900, and Potri.T158300 are listed in Supplementary Table S3. Analysis of expression levels of genes involved in cellulose biosynthesis pathways genes (*PtrCESA4*, *PtrCESA7*, and *PtrCESA8*; Suzuki et al., 2006), xylan biosynthesis pathways genes (*PtrGT43A*, *PtrGT47C*, and *PtrGT8F*; Zhou et al., 2006, 2007), lignin biosynthesis (*PtrPAL4*, *PtrC4H1*, *PtrC3H3*, *Ptr4CL5*, *PtrCCoAOMT3*, *PtrCOMT2*, *PtrCCR2*, *PtrCAld5H2*, and *PtrCAD1*), *PCD* (*PtrXCP1*, *PtrXCP2*, *PtrRNS3*, and *PtrBFN1*; Hussey et al., 2013; Cubria-Radio and Nowack, 2019), and well-known wood-associated *TFs* (*PtrWND6A*, *PtrWND6B*, *PtrMYB2*, *PtrMYB21*, *PtrMYB157*, *PtrMYB221*, *PtrMYB28*, *PtrMYB152*, *PtrNAC105*, *PtrMYB128*, *PtrMYB52*, and *PtrMYB54*) in poplars (Zhong et al., 2010a, 2011; Zhong and Ye, 2010; Hussey et al., 2013; Zhang et al., 2018b, 2020; Cubria-Radio and Nowack, 2019), were performed using qRT-PCR primers (Supplementary Table S3). *PtrActin* was employed as internal controls, and the delta-delta CT method was used to quantify gene expression levels relative to *PtrActin* (Taylor et al., 2019).

### Transient Expression Assay

The full coding region of *PtrGATA12* was cloned into pROKII under the control of the CaMV *35S* promoter as the effector construct. The reporter construct contained the β-glucuronidase (GUS) reporter gene driven by a 2-kb promoter of genes, such as *PtrWND6A*, *PtrWND6B*, *PtrMYB2*, *PtrMYB21*, *PtrMYB152*, *PtrMYB52*, *PtrCCoAOMT3*, *PtrCOMT2*, *PtrC3H3*, *PtrCAD1*, *PtrXCP1*, and *PtrGT47C*, which were chosen based on the expression analysis results of genes related to wood formation in *PtrGATA12* transgenic lines and the previous study of *AtGATA12* in *A. thaliana*, the full coding region of *PtrGATA12* was cloned into pROKII under the control of the CaMV *35S* promoter as the effector construct. The reporter construct contained the GUS reporter gene driven by a 2-kb promoter of a TF for testing, which was amplified using the primer listed in Supplementary Table S4 from the *P. trichocarpa* genomic DNA. Both effector and reporter constructs were cotransfected into tobacco leaves by *Agrobacterium tumefaciens*-mediated transient transformation (Ji et al., 2014). After agroinfiltration, plants were covered with a transparent plastic cover and transferred into a growth chamber at 25°C with 16/8 h light/dark cycle for 3 days. The transfection leaves were soaked with 100 mM MG-132 (Wako Pure Chemical) for 6 h. Then, the total protein of leaves was extracted in the extraction buffer (Yoshizumi et al., 1999). The GUS activity was measured using the 4-MUG (4-methylumbelliferyl-β-D-glucuronide) assay, and was estimated as the mean of three independent assays.

### Analysis of the Downstream *cis*-Elements of the *PtrGATA12*

Three tandem copies of the secondary NAC binding element (SNBE; Zhong et al., 2010b; McCarthy et al., 2011) and secondary MYB responding element (SMRE; Zhong et al., 2010b; Supplementary Table S5) were inserted into pHIS2 (Clontech) upstream of the reporter HIS3, respectively. The full CDS of *PtrGATA12* amplified used primers in Supplementary Table S4 was inserted into pGADT7-Rec2 as the effector vector. Both constructs were co-transformed into Y187 yeast cells, which were plated onto TOD plus 60 mM 3-amonotrizole at 30°C for 3–5 days. The cotransformation of pGADT7-rec2-p53 and pHIS2-p53 were used as a positive control, and the pGADT7-rec2-PtrGATA12 and pHIS2-p53were used as a negative control. The interactions of these sequences with the *PtrGATA12* were studied using yeast one-hybrid analysis as aforementioned. The identification of these motifs in the putative target genes promoter regions used ExactSearch tool (Gunasekara et al., 2016).

### Statistical Analysis

The Dunnett’s test (SPSS 17.0) was used to test statistical significance of data. Difference between two groups of data for comparisons in this study were evaluated by statistical significance (*, 0.01 < *p* < 0.05) or very significance (**, *p* < 0.01).

## Results

### Isolation and Characterization of *P. trichocarpa* Ortholog of *AtGATA12*

**Figure 1:**
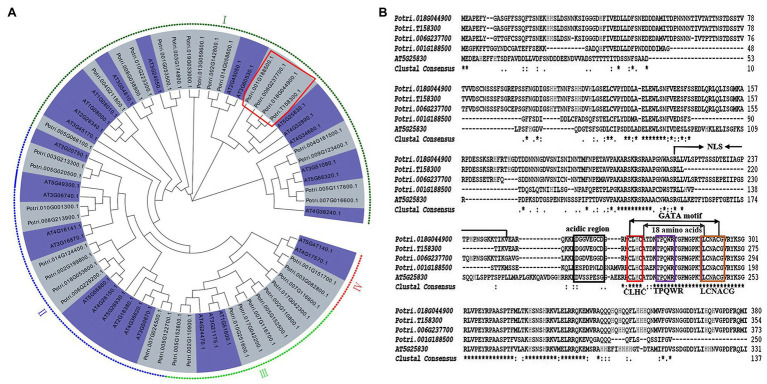
Phylogenetic analysis and protein sequence alignment of PtrGATA12. **(A)** Phylogenetic analysis of PtrGATA12 with other GATA proteins in *Populus trichocarpa* and *Arabidopsis thaliana*. PtrGATA12 proteins were shown in a red rectangle. The tree showed four major phylogenetic subfamilies (subfamilies I to IV) indicated with different colored backgrounds. **(B)** AtGATA12 protein (AT5G25830) of *A. thaliana* was aligned with PtrGATA12 proteins from *P. trichocarpa* (Potri.001G188500, Potri.006G237700, Potri.018G044900, and Potri.T158300). The conserved CLBC, TPQWR, and LCNACG in GATA motif are indicated by red box, blue box, and brown box, respectively; NLS, the putative domains for nuclear localization signals; the boxed region indicates the putative activation domain.

To identify *P. trichocarpa* ortholog of *AtGTAG12* (AT5G25830), a combined phylogenetic tree was first generated using 39 PtrGATA proteins and 30 AtGATA proteins. According to the results of phylogenetic analysis of *GATA*s in *A. thaliana* (Reyes et al., 2004), we also divided 39 *PtrGATAs* into four subgroups (I, II, III, and IV), which had 18, 10, 9, and 2 *PtrGATA* members, respectively (Figure 1A). Among the subgroup I, four poplar proteins, Potri.001G188500, Potri.006G237700, Potri.018G044900, and Potri.T158300, shared a clade with AtGTAG12, suggesting that there were four poplar orthologs of *AtGTAG12* (Figure 1A).

The multiple alignments revealed that these four PtrGATA proteins exhibited 86, 96, 96, and 96% similarity of amino acid sequence with AtGTAG12, respectively. Moreover, these PtrGATA proteins, which contain the integrated conserved GATA domain according to the zinc finger configuration C-x_2_-C-x_18_-C-x_2_-C, have the following features: (1) the presence of two pairs of Cys residues within the predicted zinc finger domain that are each separated by two amino acids (CLHC); (2) a loop of 18 amino acids between the secondary and third Cys residues; (3) conservation of the amino acid sequence LCNACG around the second Cys pair; and (4) the presence of conserved TPQWR motifs within the X_18_loop (Figure 1B). In addition, these PtrGATA proteins contained a region with 34 aa (aa211–245) that might serve as a nuclear location signal (NLS), and an acidic amino-terminal domain that might act as an activation domain (Figure 1B).

**Figure 2:**
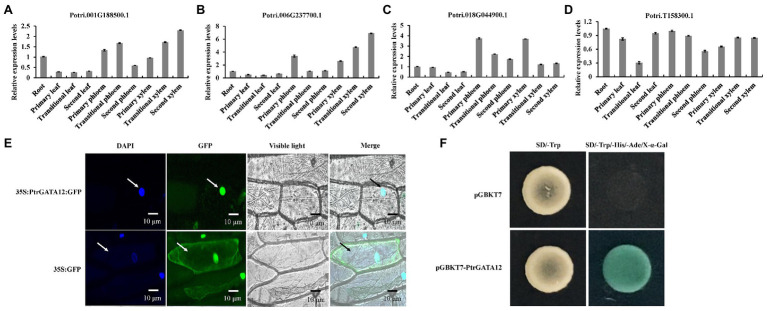
Tissue-specific expression patterns, subcellular localization and transcriptional activity of PtrGATA12. **(A)** The tissue-specific expression patterns of Potri.001G188500 gene as determined by quantitative RT-PCR (qRT-PCR) analysis. **(B)** The tissue-specific expression patterns of Potri.006G237700 gene as determined by qRT-PCR analysis. **(C)** The tissue-specific expression patterns of Potri.018G044900 gene as determined by qRT-PCR analysis. **(D)** The tissue-specific expression patterns of Potri.T158300 gene as determined by qRT-PCR analysis. *PtrActin* was used as a control. Error bars represent the SD of three biological replicates. **(E)** Subcellular localization of PtrGATA12. Confocal images manifested the localization of PtrGATA12-green fluorescent proteins (GFP) in the nuclei of onion epidermal cells. DAPI, a nuclear staining dye; Merged: the merged images of bright-field, GFP, and DAPI staining. Arrows indicate cells located in the epidermis of onions. **(F)** Transcriptional activation analysis of PtrGATA12 fused with the GAL4 DNA binding domain (GAL4DB) in yeast shows its potential to activate the expression of the His-3 and X-α-Gal reporter genes.

To determine which of these four *PtrGATAs* had the similar functions as *ATGATA12* in the wood formation, we further analyzed their expression levels in various tissues of poplar by qRT-PCR. The result showed that these four genes were differently expressed at detectable levels in all examined tissues, suggesting that they would play different roles in the development of diverse tissues of poplars. Among them, the transcript levels of Potri.006G237700 in the transition and secondary xylem were higher than those in any other studied tissues or those of other three *PtrGATA* transcripts in the same tissues. This preferential expression in developing secondary xylem tissues suggests that Potri.006G237700 is most likely to be involved in SCW biosynthesis among these four genes as *ATGATA12* is (Figures 2A–D). Thus, the Potri.006G237700 was given the nomenclature of *PtrGATA12*, in resemblance to *ATGATA12*, and chosen for functional characterization related to wood formation as described below.

### Subcellular Localization and Transcriptional Activation Activity of PtrGATA12

The presence of a nuclear localization signal in the PtrGATA12 may indicate that the protein is likely to localize in the nuclei (Figure 2E). To verify this, the *PtrGATA12* coding region was fused to the N-terminus of the GFP gene under the control of the cauliflower mosaic virus (CaMV) 35S promoter and transferred into onion epidermal cells using the particle gun bombardment. Localization of the fusion protein was then visualized with a fluorescence confocal microscope. As seen in Figure 2E, the PtrGATA12-GFP fusion protein was exclusively colocalized to DAPI-stained nuclei, indicating that *PtrGATA12* encodes a nuclear-localized protein. In addition, the presence of acidic amino-terminal domain next to GATA domain in the C-terminal region suggests that PtrGATA12 is likely a transcriptional activator. To verify this, we fused PtrGATA12 with the GAL4 DNA-binding domain and test its potential to activate the reporter gene expression in yeast. It was found that PtrGATA12 could activate the expression His-3 and X-α-Gal reporter genes (Figure 2F), indicating that it is indeed a transcriptional activator. These results demonstrated that PtrGATA12 is a transcriptional activator located in nuclei.

### Alternation of Growth-Related Traits in *PtrGATA12* Transgenic Poplar

**Figure 3:**
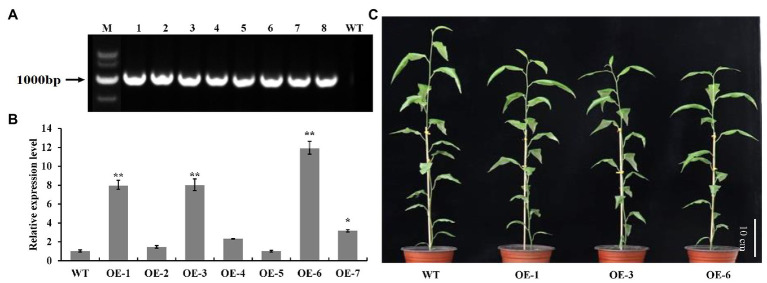
Identification and transgenic lines of *PtrGATA12* in *P. trichocarpa*. **(A)** PCR detection of *PtrGATA12* transgenic lines. M, DNA marker DL5000; 1, positive control; 2–8, PCR products of *PtrGATA12* transgenic lines; WT, wild-type *P. trichocarpa*. **(B)** qRT-PCR detection of *PtrGATA12* transgenic lines. *PtrActin* was used as a control. Each error bar represents the SD of three biological replicates. Asterisks indicate levels of significance (Dunnett’s test; *, 0.01 < *p* < 0.05, **, *p* < 0.01). **(C)** Three-month-old wild-type (WT) and *PtrGATA12* transgenic lines (OE-1, OE-3, and OE-6). Scale bars = 10 cm.

**Figure 4:**
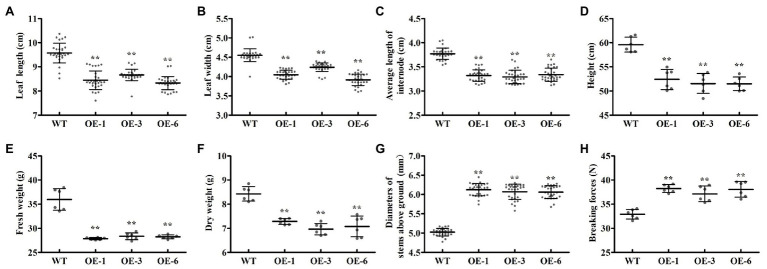
Effect of *PtrGATA12* overexpression on growth-related traits in *P. trichocarpa*. **(A–H)** Represent the leaf lengths, leaf widths, average lengths, plant heights, fresh weights, dry weights internode base diameters, and breaking forces of 90 days old WT and *PtrGATA12* transgenic lines (OE-1, OE-3, and OE-6), respectively. Each error bar represents the SD of three biological replicates. Asterisks indicate levels of significance (Dunnett’s test; **, *p* < 0.01).

To investigate the biological roles of *PtrGATA12*, we expressed *PtrGATA12* under the control of the 35S promoter in wild-type (WT) *P. trichocarpa*. In total, seven transgenic poplar lines, which exhibited normal phenotype as WT, were generated and corroborated to harbor the transformed *PtrGATA12* by genomic PCR (Figure 3A). The *PtrGATA12* expression levels in these transgenic lines were then quantitatively analyzed using qRT-PCR. Three of these *PtrGATA12* transgenic lines, OE-1, OE-3, and OE-6, which had higher expression levels of *PtrGATA12* than other transgenic lines, were chosen for further characterization (Figure 3B). Although these three *PtrGATA12* transgenic lines did not exhibit abnormal phenotypes, the growth potentials of them were slightly attenuated compared with WT (Figure 3C). For example, the leaf lengths and widths, internode lengths, and heights of *PtrGATA12* transgenic lines were reduced by 11.4 and 10.3, 12.0, and 13.4% on average than those of the WT, respectively (Figures 4A–D). The fresh and dry weights decreased by 21.7 and 15.6% on average in *PtrGATA12* transgenic lines than in WT, respectively (Figures 4E,F). In addition, it was worth noting that the diameters of stems above ground and breaking forces of stems increased by 21.0 and 14.9% on average in *PtrGATA12* transgenic lines compared with WT, respectively (Figures 4G,H). These results indicated that *PtrGATA12* overexpression may to some extent repress vegetative growth and decrease biomass for transgenic lines.

### Changes in the Characteristics of Wood Caused by *PtrGATA12* Overexpression in *P. trichocarpa*

**Figure 5:**
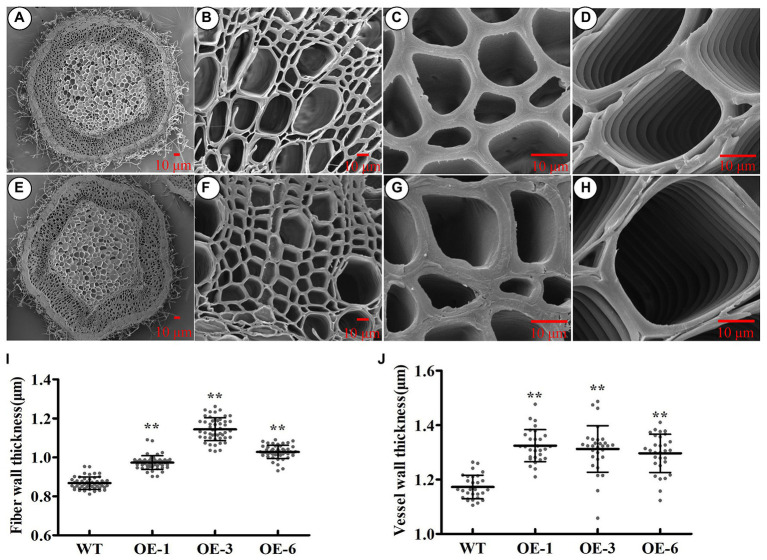
Effect of *PtrGATA12* overexpression on the secondary wall thickness of stems in *P. trichocarpa*. **(A–H)** The scanning electron microscope of cross stem sections of 90 days old wild-type **(A–D)** and *PtrGATA12* transgenic lines **(E–H)**. Scale bars = 10 μm. **(C,G)** The scanning electron microscope of fiber walls in wild-type and *PtrGATA12* transgenic lines. **(D,H)** The scanning electron microscope of vessel walls in wild-type and *PtrGATA12* transgenic lines. **(I)** The fiber wall thickness of cross stem sections in WT and *PtrGATA12* transgenic lines (OE-1, OE-3, and OE-6). **(J)** The vessel wall thickness of cross stem sections in WT and *PtrGATA12* transgenic lines. Error bars represent SD of three biological replicates. Asterisks indicate levels of significance (Dunnett’s test; *, 0.01 < *p* < 0.05, **, *p* < 0.01).

To investigate whether *PtrGATA12* had similar function as *AtGATA12* does in wood formation, we analyzed the wood characteristics in the bottom stems of the aforementioned three *PtrGATA12* transgenic lines. The scanning electron microscope of stem cross-sections revealed that the SCW thicknesses of fiber and vessel increased by 20.83 and 11.83% on average in the *PtrGATA12* transgenic lines than those of WT, respectively (Figures 5A–J).

**Figure 6:**
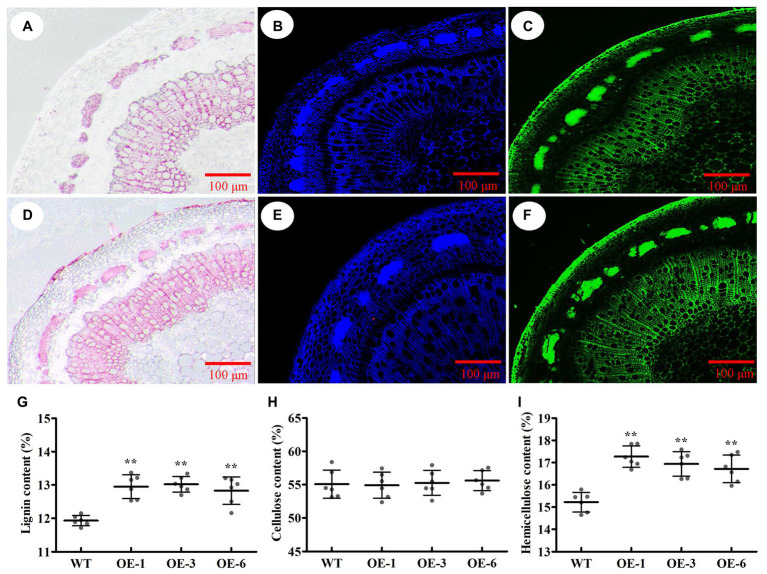
Effects of *PtrGATA12* overexpression on components of secondary cell wall of stems in *P. trichocarpa*. **(A,D)** Phloroglucinol-HCl staining (red color) stem sections of 90 days old wild-type **(A)** and *PtrGATA12* transgenic lines **(D)**. **(B,E)** Calcofluor white staining (blue color) stem sections of wild-type **(B)** and *PtrGATA12* transgenic lines **(E)**. **(C,F)** Monoclonal antibody LM10 (green color) stem sections of wild-type **(C)** and *PtrGATA12* transgenic lines **(F)**. Scale bars = 100 μm. **(G)** The lignin content of stems in WT and *PtrGATA12* transgenic lines (OE-1, OE-3, and OE-6). **(H)** The cellulose content of stems in WT and *PtrGATA12* transgenic lines. **(I)** The hemicellulose content of stems in WT and *PtrGATA12* transgenic lines. Error bars represent SD of three biological replicates. Asterisks indicate levels of significance (Dunnett’s test; **, *p* < 0.01).

To identify which component (e.g., cellulose, lignin, or hemicellulose) contributed to SCW thickening, the phloroglucinol-HCl and calcofluor white were used to stain lignin and cellulose, respectively, and the monoclonal antibody LM10 was used to label xylan immunologically. As a result, the intensities of phloroglucinol-HCl and antibody LM10 were more striking in *PtrGATA12* transgenic lines than in WT, while the intensities of calcofluor white staining were only slightly stronger in *PtrGATA12* transgenic lines than in WT (Figures 6A–F). These results demonstrated that the lignin and hemicellulose contents increased while the cellulose deposition had no obvious increase in the *PtrGATA12* transgenic lines than in WT.

To accurately assess these alternations of SCW compositions, the chemical analysis of SCW compositions was conducted using an automatic fiber analyzer and the results showed 11.5 and 8.4% increase in hemicellulose and lignin contents, respectively, and no-significant increases of cellulose content in *PtrGATA12* transgenic lines as compared to the WT (Figures 6G–I). Taken together, these results implicated that *PtrGATA12* is involved in regulating biosynthesis of some components especially lignin and hemicellulose of SCW.

### Wood Formation Pathway Genes and *TFs* Affected by *PtrGATA12* Overexpression in Poplar

**Figure 7:**
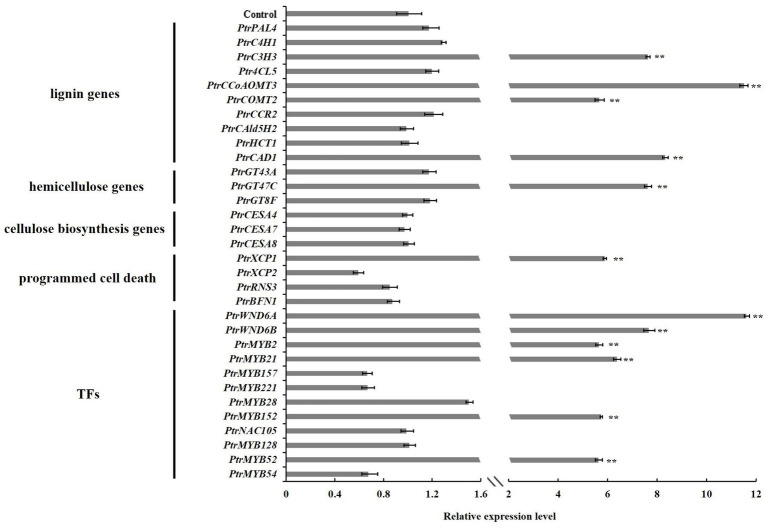
Expression analysis of wood formation pathway genes and their regulatory *TFs* in 90 days old wild-type and *PtrGATA12* transgenic lines. Control represents the normalized expression level (namely 1 in this case) of each wood formation gene examined in wild-type plants. Error bars represent SD of three biological replicates. Asterisks indicate levels of significance (Dunnett’s test; **, *p* < 0.01).

To investigate the molecular basis of the alternations of wood characteristics described above, we further studied the expressions of genes related to SCW biosynthesis in the stems of *PtrGATA12* transgenic lines. The results from qRT-PCR analysis demonstrated that the transcript levels of lignin biosynthesis genes, including *PtrC3H3*, *PtrCCoAOMT3*, *PtrCOMT2*, and *PtrCAD1*, and xylan biosynthesis genes, such as *PtrGT47C*, were significantly increased in the *PtrGATA12* transgenic lines compared with the WT (Figure 7). On the contrary, the cellulose biosynthesis genes, such as *PtrCESA4*, *PtrCESA7*, and *PtrCESA8*, exhibited no obvious upregulation in *PtrGATA12* transgenic lines compared with WT. In addition, it is worth mentioning that *PtrXCP1*, whose counterpart in *A. thaliana* is specifically expressed in xylem tissues at the onset of PCD, was also significantly upregulated in *PtrGATA12* transgenic lines compared with WT (Figure 7).

Given the fact that *AtGATA12* is an upstream *TF* of *AtVND7* (Endo et al., 2015), we investigated whether *PtrGATA12* could affect the expressions of poplar orthologs of *AtVND7*, *PtrWND6A*, and *PtrWND6B* as well as other well-known wood-associated *TFs* in transgenic poplar. The qRT-PCR analysis revealed that the transcript abundances of master switches, such as *PtrWND6A*, *PtrWND6B*, *PtrMYB2*, and *PtrMYB21* (Zhang et al., 2018b, 2020), were significantly upregulated in *PtrGATA12* transgenic lines compared with WT (Figure 7). In addition, the *TFs* specifically activating lignin biosynthesis, such as *PtrMYB152*, were significantly upregulated in *PtrGATA12* transgenic lines compared with WT. The expression levels of *PtrNAC105* and *PtrMYB128* involved in activation of cellulose biosynthesis (Zhang et al., 2020) showed no obvious increases in *PtrGATA12* transgenic lines compared with WT (Figure 7). Moreover, the TFs related to activation of PCD, such as *PtrMYB52* (Zhang et al., 2018b), was significantly upregulated in *PtrGATA12* transgenic lines compared with WT. These results are aligned well with the alternations of SCW characteristics observed in *PtrGATA12* transgenic lines.

### Regulation of Poplar *TFs* and Pathway Genes Involved in Wood Formation by PtrGATA12 in Tobacco

**Figure 8:**
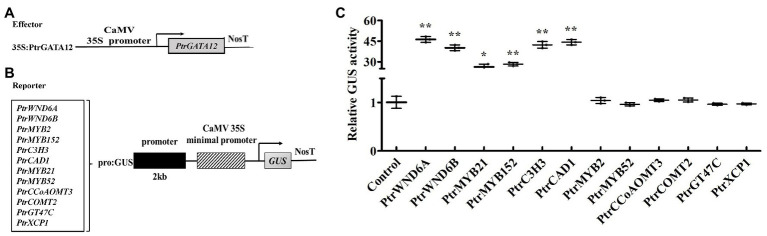
Activation or repression of the promoters of poplar transcription factors and secondary cell wall (SCW) pathway genes by PtrGATA12. **(A,B)** Diagrams of the effector and reporter constructs. **(C)** The expression of the β-glucuronidase (GUS) under the control of those promoters by PtrGATA12. GUS activity in tobacco leaves transfected with the reporter construct alone was used as a control and was set to 1. Error bars represent SD of three biological replicates. Asterisks indicate levels of significance of differential expression (Dunnett’s test; *, 0.01 < *p* < 0.05, **, *p* < 0.01).

Based on fact that *PtrGATA12* significantly altered the expression of a number of wood-associated genes in the poplar transgenic lines, we investigated whether PtrGATA12 can directly control these genes using a transient expression system in tobacco. We selected those *TFs* and pathway genes whose expression had a fold change of more than five times in *PtrGATA12* poplar transgenic lines compared with WT for testing. The 2-kb proximal promoter regions of these genes amplified from *P. trichocarpa* genomic DNA were linked to the GUS reporter gene to create the reporter constructs, and the full-length cDNA of *PtrGATA12* was ligated to 35S promoter to generate the effector construct (Figures 8A,B). The reporter and effector constructs were co-transfected into tobacco leaves by *Agrobacterium*-mediated method. The subsequent assay of the GUS activity in the transfected leaves demonstrated that PtrGATA12 significantly activated the activities of *PtrWND6A*, *PtrWND6B*, *PtrMYB21*, and *PtrMYB152* promoters (Figure 8C), albeit to different levels. It was notable that PtrGATA12 significantly activated the activities of promoters of lignin biosynthesis genes, *PtrC3H3* and *PtrCAD1* (Figure 8C). However, PtrGATA12 had no regulatory effects on the activities of *PtrMYB2*, *PtrMYB52*, *PtrCCoAOMT3*, *PtrCOMT2*, *PtrXCP1*, and *PtrGT47C* promoters (Figure 8C). These results suggested that *PtrGATA12* could directly control some *TFs* and pathway genes during SCW biosynthesis.

### The Potential of PtrGATA12 as a Higher Hierarchical TF

**Figure 9:**
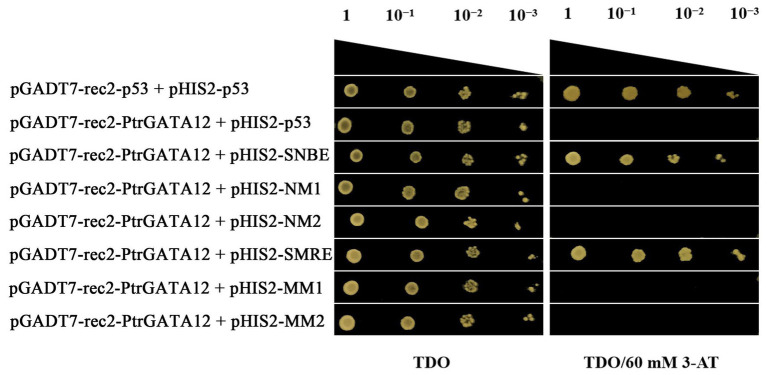
Yeast one-hybrid assay of PtrGATA12 binding to *cis*-acting elements. The pGADT7-rec2-p53/pHIS2-p53 and pGADT7-rec2-PtrGATA12/pHIS2-p53 were used as the positive and negative control, respectively. 10^−1^, 10^−2^, and 10^−3^ represent solution dilution ratio of transformed Y187 yeast cells.

To test whether *PtrGATA12* directly regulated these aforementioned genes through binding to the *cis*-acting elements in their promoters, a yeast one-hybrid assay was performed to test the potential of PtrGATA12 binding to SNBE and SMRE *cis*-acting elements (Supplementary Table S5), which are often present in the promoters of those genes that are directly controlled by PtrGATA12 (Supplementary Table S6) and those that are components of the currently known hierarchical transcriptional network of secondary wall formation (Ye and Zhong, 2015). The results manifested that PtrGATA12 had obvious binding affinities to these *cis*-acting (Figure 9), which demonstrated that PtrGATA12 as a higher hierarchical *TF* could bind to specific *cis*-acting elements to directly control *TFs* at low hierarchy and pathway genes during SCW biosynthesis.

## Discussion

In the last decade, considerable studies have revealed that the SCW biosynthesis is mainly regulated by a multilayered hierarchical transcription regulation network with the regulatory edges, chains of command, pointing downward the pathway genes at the low hierarchy, which ensures a differential regulation on the formation of diverse SCW with varied components and thicknesses in various cells and across different tissues (Zhong et al., 2007, 2010a; Zhou et al., 2009; Du and Groover, 2010; Yamaguchi et al., 2010). Generally speaking, the regulators with higher hierarchy are more likely serve as master regulators or the regulatory switches exerting different regulation on multiple pathways (Balazsi et al., 2005). For example, in *P. trichocapa*, a well-characterized higher hierarchical regulator is *PtrSND1-B1*, which mediates a four-layered network (Chen et al., 2019). In this network, PtrSND1-B1 directly regulates *Ptr-MYB021* and *PtrMYB074*, whose proteins, in turn, regulate 15 *TFs*, *PtrNAC127*, *PtrMYB175*, *PtrWBLH3*, *PtrHAM3*, *PtrMYB059*, *PtrMYB090*, *PtrMYB161*, *PtrMYB174*, *PtrNAC123*, *PtrWBLH*, *PtrWBLH2*, *PtrMYB088*, *PtrMYB093*, *PtrNAC105*, and *PtrNAC126*; these 15 *TFs* then directly control some lignin biosynthetic genes, *PtrCAD1*, *PtrHCT1*, *PtrHCT6*, *PtrC4H1*, *PtrCOMT2*, *PtrCCoAOMT1,2*, and *PtrCAld5H1,2*, and three cellulose synthases, *PtrCesA4/17/18*. Chen et al. (2019) also characterized the *AtSND1*-mediated network in *A. thaliana* in their study, and found that it has only three layers and is very different from *PtrSND1-B1*-mediated network in poplars. *AtSND1* directly regulates 14 *TFs* (including at *AtMYB46*, a counterpart of *PtrMYB21*; Ezcurra et al., 2011) and seven cell wall biosynthetic genes, and *AtMYB46* regulates 17 *TFs* and 12 SCW biosynthetic genes (including eight lignin pathway genes). In our study, we found that the *PtrGATA12* was a regulator of *PtrMYB21*, a target gene of *PtrSND1-B1* too, indicating that *PtrGATA12* is at the same or higher hierarchical level than that of *PtrSND1-B1*. This is because: (1) *PtrMYB21* was significantly upregulated more than five times in *PtrGATA12* transgenic lines as compared to WT (Figure 7). (2) The yeast one-hybrid assay also confirmed that PtrGATA12 could bind the two SMRE motifs (ACCAACT) and SNBE motif (AACCTTGAGTTTGAAGGAT; Figure 9) and activated the reporter genes in yeast. Both motifs are present in in the 2 kb promoter of *PtrMYB21* (Supplementary Table S6). (3) The overexpression of *PtrGATA12* using the transient GUS-activation system in tobacco lead to a significant upregulation of *PtrMYB21*, which may indicate that PtrGATA12 could directly activate *PtrMYB21*. Collectively, these results demonstrate and suggest that PtrGATA12 is higher hierarchical regulator than *PtrMYB21*.

In addition to *PtrMYB21*, our study using GUS activation system also demonstrated that *PtrWND6A* and *PtrWND6B* are target genes of *PtrGATA12* and their promoter regions (2 kb) contain both SNBE and SMRE motifs (Supplementary Table S6). These two genes are most phylogenetically related to *VND7* (Ye and Zhong, 2015), which is a higher hierarchical regulator/switch regulating xylem vessel differentiation of protoxylem vessels in *Arabidopsis* (Endo et al., 2015). *PtrWNDs* have been indicated to serve as higher hierarchical regulators of not only wood associated MYB master switches (e.g., *PtrMYB2*, *3*, *20*, and *21*), but also *TFs* activating all three SCW biosynthesis pathways (e.g., *PtrMYB128*, *PtrNAC150*, *PtrMYB75*, and *PtrMYB18*; Ye and Zhong, 2015). Our transient GUS-activation system in tobacco and yeast one-hybrid assays analysis revealed that PtrGATA12 could directly activate *PtrWND6A/B*, *PtrMYB21*, *PtrMYB152*, *PtrC3H3*, and *PtrCAD1* through binding to SNBE, and SMRE *cis*-elements present in their promoter regions (Figures 8C, 9). These results explicitly suggest that *PtrGATA12* modulate SCW biosynthesis pathways through directly regulating some high hierarchical regulators including *PtrWND6A*, *PtrWND6B*, and *PtrMYB21*, and indirectly regulating well-known wood-associated *TFs* and pathway genes that are elements of transcription regulation network of poplar SCW formation. In line with alternations of SCW components, the expression of pathway genes related to lignin and xylan biosynthesis and PCD exhibited significant changes in *PtrGATA12* poplar transgenic lines (Figure 7). Besides these, the expressions of *TFs* in different layers of hierarchical transcription regulation network, such as *PtrMYB28*, *PtrMYB157*, *PtrMYB221*, *PtrMYB52*, and *PtrMYB54* (Mellerowicz and Sundberg, 2008; Zhong et al., 2008, 2010a, 2011; Zhong and Ye, 2010; Taylor-Teeples et al., 2015; Rao and Dixon, 2018), also exhibited obvious alternations in *PtrGATA12* poplar transgenic lines (Figure 7). As a result, *PtrGATA12* switched on hemicellulose and lignin pathway while maintaining the cellulose biosynthesis largely unchanged.

Previous study also showed that *A. thaliana TF*, *SHINE2* (*SHN2*), downregulates the lignin biosynthesis while upregulating the cellulose and hemicellulose biosynthesis in rice (Ambavaram et al., 2011). This is largely opposite to the biological function of *PtrGATA12* in SCW formation. Based on the experimental evidence, a hypothetical model was established assuming that *SHN2* serves as a hierarchical regulator that downregulate lignin biosynthetic genes through repressing *MYB58/63*, whereas upregulate the cellulose biosynthesis through activating *MYB20/43*. We previously overexpressed *Populus simonii* × *Populus nigra PsnSHN2* in tobacco, leading to the altered the SCW characteristics, of which the most significant changes were the contents of cellulose and hemicellulose that increased 37 and 28%, respectively, whereas the content of lignin that decreased 34% (Liu et al., 2017). But, unfortunately, we did not test if *PtrGATA12* regulate these of *PsnSHN2’s* target *TFs* in this study. Our study showed that *PtrGATA12* regulated SCW pathways through possibly regulating *PtrWND6A*, *PtrWND6B*, *PtrMYB21*, and *PtrMYB152*, whose family members also showed in *PsnSHN2* down-stream target *TFs*, for example, *PsnWND1A*, *PsnWND3A*, *PsnMYB3*, and *PsnMYB152*. These evidences and the differential regulation of *PtrGATA12* and *PsnSHN2* on SCW component biosynthetic pathways indicate that *PtrGATA12* is just another very valuable high hierarchical regulator like *PsnSHN2*, and can be used alone or in conjunction with *PsnSHN2* in genetic engineering of woody plants to produce the wood with altered SCW components to meet a variety of needs.

Besides alternations of SCW characteristics, the *PtrGATA12* transgenic lines exhibited arrested growths of most phenotype traits and biomass accumulation (Figures 3C, 4), which was consistent with previous reports that the growth of plants often has strong inverse correlation with lignin accumulation (Novaes et al., 2009, 2010). In addition, the breaking forces of *PtrGATA12* transgenic lines became stronger than those of WT (Figure 4H). The previous studies have revealed that the tensile or bending strength of plant stems is positively correlated with the cellulose content (Dhugga, 2007; Voelker et al., 2011; Liu et al., 2017), which did not exhibit significant increases in *PtrGATA12* transgenic lines than in WT (Figure 6H), and thus was not the major contributor to the increased breaking forces in *PtrGATA12* transgenic lines. We have noticed that the *PtrGATA12* transgenic lines exhibited significant increases in diameters of stems (Figure 4C), SCW thickness of fibers and vessels (Figure 5), and hemicellulose content of SCW (Figure 6I), which might be responsible for the breaking forces of stem increased in *PtrGATA12* transgenic lines. These changes also support that *PtrGATA12*, as a higher hierarchical regulator, had pleiotropic effects on SCW biosynthesis and vegetative growth in poplars through regulating multiple biological pathways in wood development.

In summary, our study demonstrated that *PtrGATA12*, as a higher hierarchical regulator, differentially modulates multiple SCW component biosynthetic pathways through possibly regulating multiple master regulators including *PtrWND6A*, *PtrWND6B*, *PtrMYB21*, and *PtrMYB152*. It can be specifically used for augmenting lignin and hemicellulose biosynthesis without reducing cellulose.

## Data Availability Statement

The raw data supporting the conclusions of this article will be made available by the authors, without undue reservation.

## Author Contributions

MR and YZ conducted most experiments and data analysis. CL and YL participated in the histochemical staining. ST and HC participated in the vegetative propagation of *Populus trichocarpa* plantlets and phenotype analysis. HZ reviewed the manuscript. HW performed the data analysis and wrote the manuscript. ZW designed the experiments and wrote the manuscript. All authors read and approved the final version of the manuscript.

### Conflict of Interest

The authors declare that the research was conducted in the absence of any commercial or financial relationships that could be construed as a potential conflict of interest.

## Footnotes

^1^http://rsbweb.nih.gov/ij/

## References

[ref1] Ambavaram M. M. Krishnan A. Trijatmiko K. R. Pereira A. (2011). Coordinated activation of cellulose and repression of lignin biosynthesis pathways in rice. Plant Physiol. 155, 916–931. 10.1104/pp.110.168641, PMID: 21205614 PMC3032476

[ref2] An Y. Han X. Tang S. Xia X. Yin W. (2014). Poplar GATA transcription factor PdGNC is capable of regulating chloroplast ultrastructure, photosynthesis, and vegetative growth in *Arabidopsis* under varying nitrogen levels. Plant Cell Tissue Organ Cult. 119, 313–327. 10.1007/s11240-014-0536-y

[ref3] An Y. Zhou Y. Y. Han X. Shen C. Wang S. Liu C.. (2020). The GATA transcription factor GNC plays an important role in photosynthesis and growth in poplar. J. Exp. Bot. 71, 1969–1984. 10.1093/jxb/erz564, PMID: 31872214 PMC7094078

[ref4] Balazsi G. Barabasi A. L. Oltvai Z. N. (2005). Topological units of environmental signal processing in the transcriptional regulatory network of *Escherichia coli*. Proc. Natl. Acad. Sci. U. S. A. 102, 7841–7846. 10.1073/pnas.0500365102, PMID: 15908506 PMC1142363

[ref5] Balmant K. M. Noble J. D. Alves F. C. Dervinis C. Conde D. Schmidt H. W.. (2020). Xylem systems genetics analysis reveals a key regulator of lignin biosynthesis in *Populus deltoides*. Genome Res. 30, 1131–1143. 10.1101/gr.261438.120, PMID: 32817237 PMC7462072

[ref6] Behringer C. Bastakis E. Ranftl Q. L. Mayer K. F. Schwechheimer C. (2014). Functional diversification within the family of B-GATA transcription factors through the leucine-leucine-methionine domain. Plant Physiol. 166, 293–305. 10.1104/pp.114.246660, PMID: 25077795 PMC4149714

[ref7] Behringer C. Schwechheimer C. (2015). B-GATA transcription factors—insights into their structure, regulation, and role in plant development. Front. Plant Sci. 6:90. 10.3389/fpls.2015.00090, PMID: 25755661 PMC4337238

[ref8] Chen H. F. Shao H. X. Li K. Zhang D. Fan S. Li Y. M.. (2017). Genome-wide identification, evolution, and expression analysis of GATA transcription factors in apple (Malus x domestica Borkh.). Gene 627, 460–472. 10.1016/j.gene.2017.06.049, PMID: 28669931

[ref9] Chen H. Wang J. P. Liu H. Li H. Lin Y. J. Shi R.. (2019). Hierarchical transcription factor and chromatin binding network for wood formation in black cottonwood (*Populus trichocarpa*). Plant Cell 31, 602–626. 10.1105/tpc.18.00620, PMID: 30755461 PMC6482634

[ref10] Cubria-Radio M. Nowack M. K. (2019). Transcriptional networks orchestrating programmed cell death during plant development. Curr. Top. Dev. Biol. 131, 161–184. 10.1016/bs.ctdb.2018.10.006, PMID: 30612616 PMC7116394

[ref11] Dhugga K. S. (2007). Maize biomass yield and composition for biofuels. Crop Sci. 47, 2211–2227. 10.2135/cropsci2007.05.0299

[ref12] Du J. Groover A. (2010). Transcriptional regulation of secondary growth and wood formation. J. Integr. Plant Biol. 52, 17–27. 10.1111/j.1744-7909.2010.00901.x, PMID: 20074137

[ref13] Endo H. Yamaguchi M. Tamura T. Nakano Y. Nishikubo N. Yoneda A.. (2015). Multiple classes of transcription factors regulate the expression of VASCULAR-RELATED NAC-DOMAIN7, a master switch of xylem vessel differentiation. Plant Cell Physiol. 56, 242–254. 10.1093/pcp/pcu134, PMID: 25265867

[ref14] Ezcurra I. Johansson C. Tamizhselvan P. Winzell A. Aspeborg H. (2011). An AC-type element mediates transactivation of secondary cell wall carbohydrate-active enzymes by PttMYB021, the *Populus* MYB46 orthologue. BMC Proc. 5(Suppl. 7):O40. 10.1186/1753-6561-5-s7-o40

[ref15] Gunasekara C. Subramanian A. Avvari J. V. Li B. Chen S. Wei H. (2016). ExactSearch: a web-based plant motif search tool. Plant Methods 12:26. 10.1186/s13007-016-0126-6, PMID: 27134638 PMC4850730

[ref16] Hussey S. G. Mizrachi E. Creux N. M. Myburg A. A. (2013). Navigating the transcriptional roadmap regulating plant secondary cell wall deposition. Front. Plant Sci. 4:325. 10.3389/fpls.2013.00325, PMID: 24009617 PMC3756741

[ref17] Ji X. Y. Zheng L. Liu Y. J. Nie X. G. Liu S. N. Wang Y. C. (2014). A transient transformation system for the functional characterization of genes involved in stress response. Plant Mol. Biol. Report. 32, 732–739. 10.1007/s11105-013-0683-z

[ref19] Ko J. H. Kim W. C. Kim J. Y. Ahn S. J. Han K. H. (2012). MYB46-mediated transcriptional regulation of secondary wall biosynthesis. Mol. Plant 5, 961–963. 10.1093/mp/sss076, PMID: 22914575

[ref20] Kobayashi K. Masuda T. (2016). Transcriptional regulation of tetrapyrrole biosynthesis in *Arabidopsis thaliana*. Front. Plant Sci. 7:1811. 10.3389/fpls.2016.01811, PMID: 27990150 PMC5130987

[ref21] Kobayashi K. Ohnishi A. Sasaki D. Fujii S. Iwase A. Sugimoto K.. (2017). Shoot removal induces chloroplast development in roots via cytokinin signaling. Plant Physiol. 173, 2340–2355. 10.1104/pp.16.01368, PMID: 28193764 PMC5373043

[ref22] Kolosova N. Miller B. Ralph S. Ellis B. E. Douglas C. Ritland K.. (2004). Isolation of high-quality RNA from gymnosperm and angiosperm trees. BioTechniques 36, 821–824. 10.2144/04365ST06, PMID: 15152602

[ref23] Li S. F. Zhang Y. X. Xin X. B. Ding C. J. Lv F. L. Mo W. J.. (2020). The Osmotin-Like protein gene PdOLP1 is involved in secondary cell wall biosynthesis during wood formation in poplar. Int. J. Mol. Sci. 21:3993. 10.3390/ijms21113993, PMID: 32498411 PMC7312728

[ref24] Li S. Zhen C. Xu W. Wang C. Cheng Y. (2017). Simple, rapid and efficient transformation of genotype Nisqually-1: a basic tool for the first sequenced model tree. Sci. Rep. 7:2638. 10.1038/s41598-017-02651-x, PMID: 28572673 PMC5453977

[ref25] Liao Z. Chen M. Guo L. Gong Y. Tang F. Sun X.. (2004). Rapid isolation of high-quality total RNA from taxus and ginkgo. Prep. Biochem. Biotechnol. 34, 209–214. 10.1081/PB-200026790, PMID: 15461137

[ref26] Liu Y. Wei M. Hou C. Lu T. Liu L. Wei H.. (2017). Functional characterization of *Populus* PsnSHN2 in coordinated regulation of secondary wall components in tobacco. Sci. Rep. 7:42. 10.1038/s41598-017-00093-z, PMID: 28246387 PMC5428377

[ref27] Lowry J. A. Atchley W. R. (2000). Molecular evolution of the GATA family of transcription factors: conservation within the DNA-binding domain. J. Mol. Evol. 50, 103–115. 10.1007/s002399910012, PMID: 10684344

[ref28] McCarthy R. L. Zhong R. Fowler S. Lyskowski D. Piyasena H. Carleton K.. (2010). The poplar MYB transcription factors, PtrMYB3 and PtrMYB20, are involved in the regulation of secondary wall biosynthesis. Plant Cell Physiol. 51, 1084–1090. 10.1093/pcp/pcq064, PMID: 20427511

[ref29] McCarthy R. L. Zhong R. Ye Z. H. (2009). MYB83 is a direct target of SND1 and acts redundantly with MYB46 in the regulation of secondary cell wall biosynthesis in *Arabidopsis*. Plant Cell Physiol. 50, 1950–1964. 10.1093/pcp/pcp139, PMID: 19808805

[ref30] McCarthy R. L. Zhong R. Ye Z. H. (2011). Secondary wall NAC binding element (SNBE), a key cis-acting element required for target gene activation by secondary wall NAC master switches. Plant Signal. Behav. 6, 1282–1285. 10.4161/psb.6.9.16402, PMID: 21847026 PMC3258052

[ref31] Mellerowicz E. J. Sundberg B. (2008). Wood cell walls: biosynthesis, developmental dynamics and their implications for wood properties. Curr. Opin. Plant Biol. 11, 293–300. 10.1016/j.pbi.2008.03.003, PMID: 18434240

[ref32] Novaes E. Kirst M. Chiang V. Winter-Sederoff H. Sederoff R. (2010). Lignin and biomass: a negative correlation for wood formation and lignin content in trees. Plant Physiol. 154, 555–561. 10.1104/pp.110.161281, PMID: 20921184 PMC2949025

[ref33] Novaes E. Osorio L. Drost D. R. Miles B. L. Boaventura-Novaes C. R. Benedict C.. (2009). Quantitative genetic analysis of biomass and wood chemistry of *Populus* under different nitrogen levels. New Phytol. 182, 878–890. 10.1111/j.1469-8137.2009.02785.x, PMID: 19291008

[ref34] Ohashi-Ito K. Oda Y. Fukuda H. (2010). *Arabidopsis* VASCULAR-RELATED NAC-DOMAIN6 directly regulates the genes that govern programmed cell death and secondary wall formation during xylem differentiation. Plant Cell 22, 3461–3473. 10.1105/tpc.110.075036, PMID: 20952636 PMC2990123

[ref35] Ohlrogge J. Allen D. Berguson B. Dellapenna D. Shachar-Hill Y. Stymne S. (2009). Energy. Driving on biomass. Science 324, 1019–1020. 10.1126/science.1171740, PMID: 19460990

[ref36] Rao X. Dixon R. A. (2018). Current models for transcriptional regulation of secondary cell wall biosynthesis in grasses. Front. Plant Sci. 9:399. 10.3389/fpls.2018.00399, PMID: 29670638 PMC5893761

[ref37] Reyes J. C. Muro-Pastor M. I. Florencio F. J. (2004). The GATA family of transcription factors in *Arabidopsis* and rice. Plant Physiol. 134, 1718–1732. 10.1104/pp.103.037788, PMID: 15084732 PMC419845

[ref38] Rueda-Lopez M. Pascual M. B. Pallero M. Henao L. M. Lasa B. Jauregui I.. (2017). Overexpression of a pine Dof transcription factor in hybrid poplars: a comparative study in trees growing under controlled and natural conditions. PLoS One 12:e0174748. 10.1371/journal.pone.0174748, PMID: 28376100 PMC5380328

[ref39] Shikata M. Matsuda Y. Ando K. Nishii A. Takemura M. Yokota A.. (2004). Characterization of *Arabidopsis* ZIM, a member of a novel plant-specific GATA factor gene family. J. Exp. Bot. 55, 631–639. 10.1093/jxb/erh078, PMID: 14966217

[ref40] Suzuki S. Li L. Sun Y. H. Chiang V. L. (2006). The cellulose synthase gene superfamily and biochemical functions of xylem-specific cellulose synthase-like genes in *Populus trichocarpa*. Plant Physiol. 142, 1233–1245. 10.1104/pp.106.086678, PMID: 16950861 PMC1630762

[ref41] Taylor S. C. Nadeau K. Abbasi M. Lachance C. Nguyen M. Fenrich J. (2019). The ultimate qPCR experiment: producing publication quality, reproducible data the first time. Trends Biotechnol. 37, 761–774. 10.1016/j.tibtech.2018.12.002, PMID: 30654913

[ref42] Taylor-Teeples M. Lin L. de Lucas M. Turco G. Toal T. W. Gaudinier A.. (2015). An *Arabidopsis* gene regulatory network for secondary cell wall synthesis. Nature 517, 571–575. 10.1038/nature14099, PMID: 25533953 PMC4333722

[ref43] Tuskan G. A. Difazio S. Jansson S. Bohlmann J. Grigoriev I. Hellsten U.. (2006). The genome of black cottonwood, *Populus trichocarpa* (Torr. & Gray). Science 313, 1596–1604. 10.1126/science.1128691, PMID: 16973872

[ref44] Voelker S. L. Lachenbruch B. Meinzer F. C. Strauss S. H. (2011). Reduced wood stiffness and strength, and altered stem form, in young antisense 4CL transgenic poplars with reduced lignin contents. New Phytol. 189, 1096–1109. 10.1111/j.1469-8137.2010.03572.x, PMID: 21158867

[ref45] Yamaguchi M. Goue N. Igarashi H. Ohtani M. Nakano Y. Mortimer J. C.. (2010). VASCULAR-RELATED NAC-DOMAIN6 and VASCULAR-RELATED NAC-DOMAIN7 effectively induce transdifferentiation into xylem vessel elements under control of an induction system. Plant Physiol. 153, 906–914. 10.1104/pp.110.154013, PMID: 20488898 PMC2899931

[ref46] Ye Z. H. Zhong R. (2015). Molecular control of wood formation in trees. J. Exp. Bot. 66, 4119–4131. 10.1093/jxb/erv081, PMID: 25750422

[ref47] Yoshizumi T. Nagata N. Shimada H. Matsui M. (1999). An *Arabidopsis* cell cycle-dependent kinase-related gene, CDC2b, plays a role in regulating seedling growth in darkness. Plant Cell 11, 1883–1896. 10.1105/tpc.11.10.1883, PMID: 10521519 PMC144097

[ref48] Zhang C. Hou Y. Hao Q. Chen H. Chen L. Yuan S.. (2015). Genome-wide survey of the soybean GATA transcription factor gene family and expression analysis under low nitrogen stress. PLoS One 10:e0125174. 10.1371/journal.pone.0145988, PMID: 25886477 PMC4401516

[ref49] Zhang Z. Ren C. Zou L. Wang Y. Li S. Liang Z. (2018a). Characterization of the GATA gene family in *Vitis vinifera*: genome-wide analysis, expression profiles, and involvement in light and phytohormone response. Genome 61, 713–723. 10.1139/gen-2018-0042, PMID: 30092656

[ref50] Zhang J. Tuskan G. A. Tschaplinski T. J. Muchero W. Chen J. G. (2020). Transcriptional and post-transcriptional regulation of lignin biosynthesis pathway genes in *Populus*. Front. Plant Sci. 11:616977. 10.3389/fpls.2020.616977, PMID: 32528504 PMC7262965

[ref51] Zhang J. Xie M. Tuskan G. A. Muchero W. Chen J. G. (2018b). Recent advances in the transcriptional regulation of secondary cell wall biosynthesis in the woody plants. Front. Plant Sci. 9:1535. 10.3389/fpls.2018.01535, PMID: 30405670 PMC6206300

[ref52] Zhang Z. Zou X. Y. Huang Z. Fan S. M. Qun G. Liu A. Y.. (2019). Genome-wide identification and analysis of the evolution and expression patterns of the GATA transcription factors in three species of *Gossypium* genus. Gene 680, 72–83. 10.1016/j.gene.2018.09.039, PMID: 30253181

[ref53] Zhong R. Lee C. Ye Z. H. (2010a). Functional characterization of poplar wood-associated NAC domain transcription factors. Plant Physiol. 152, 1044–1055. 10.1104/pp.109.148270, PMID: 19965968 PMC2815876

[ref54] Zhong R. Lee C. Ye Z. H. (2010b). Global analysis of direct targets of secondary wall NAC master switches in *Arabidopsis*. Mol. Plant 3, 1087–1103. 10.1093/mp/ssq062, PMID: 20935069

[ref55] Zhong R. Lee C. Zhou J. McCarthy R. L. Ye Z. H. (2008). A battery of transcription factors involved in the regulation of secondary cell wall biosynthesis in *Arabidopsis*. Plant Cell 20, 2763–2782. 10.1105/tpc.108.061325, PMID: 18952777 PMC2590737

[ref56] Zhong R. McCarthy R. L. Lee C. Ye Z. H. (2011). Dissection of the transcriptional program regulating secondary wall biosynthesis during wood formation in poplar. Plant Physiol. 157, 1452–1468. 10.1104/pp.111.181354, PMID: 21908685 PMC3252164

[ref57] Zhong R. Richardson E. A. Ye Z. H. (2007). The MYB46 transcription factor is a direct target of SND1 and regulates secondary wall biosynthesis in *Arabidopsis*. Plant Cell 19, 2776–2792. 10.1105/tpc.107.053678, PMID: 17890373 PMC2048704

[ref58] Zhong R. Ye Z. H. (2010). The poplar PtrWNDs are transcriptional activators of secondary cell wall biosynthesis. Plant Signal. Behav. 5, 469–472. 10.4161/psb.5.4.11400, PMID: 20383071 PMC2958599

[ref59] Zhou J. Lee C. Zhong R. Ye Z. H. (2009). MYB58 and MYB63 are transcriptional activators of the lignin biosynthetic pathway during secondary cell wall formation in *Arabidopsis*. Plant Cell 21, 248–266. 10.1105/tpc.108.063321, PMID: 19122102 PMC2648072

[ref60] Zhou G. K. Zhong R. Q. Himmelsbach D. S. McPhail B. T. Ye Z. H. (2007). Molecular characterization of PoGT8D and PoGT43B, two secondary wall-associated glycosyltransferases in poplar. Plant Cell Physiol. 48, 689–699. 10.1093/pcp/pcm037, PMID: 17379696

[ref61] Zhou G. K. Zhong R. Q. Richardson E. A. Morrison W. H. Nairn C. J. Wood-Jones A.. (2006). The poplar glycosyltransferase GT47C is functionally conserved with *Arabidopsis* fragile fiber8. Plant Cell Physiol. 47, 1229–1240. 10.1093/pcp/pcj093, PMID: 16887843

